# Supplementary material for: Fixing the stimulus-as-fixed-effect fallacy in task fMRI
Source: Wellcome Open Res. 2017 Mar 17;1:23. Originally published 2016 Dec 9. [Version 2] doi: 10.12688/wellcomeopenres.10298.2 (PMC5428747; doi:10.12688/wellcomeopenres.10298.2)
Supplement: Supplementary file 1 [file wellcomeopenres-1-11977-s0000.tgz › 74653d11-2215-4d86-8256-22ec69deb4e1.pdf]

Statistical models

As mentioned in the main text, we implemented the Random Stimulus Model (RSM) as a Bayesian model, primarily in order to circumvent certain limitations in standard statistical software for fitting linear mixed models. In order to facilitate comparisons between the RSM and the standard model applied in most research papers, we also implemented the standard model as a Bayesian model, so that the two models differed only in their inclusion of random stimulus effects. Here we give the full Bayesian mixed models that we used for all 6 datasets we reanalyzed. Each model contains a set of stimulus-level regressors x_{ijt} that, as in the main text, represent the predicted neural response of the ith subject toward the jth stimulus at the tth time point. These regressors were constructed by convolving a stimulus presentation sequence, indicating when a stimulus was being presented, with a double-gamma hemodynamic response function (HRF) with parameters fixed at the default values used in the SPM package.

For each task, the model was applied separately to 100 regions-of-interest (ROIs) defined via meta-analytic k-means clustering of over 11,000 studies in the Neurosynth dataset (http://neurosynth.org; details are reported in a forthcoming publication). The 100-ROI whole-brain cluster map is available from the project GitHub repository (http://github.com/PsychoinformaticsLab/nipymc). Note that this mask was used purely for convenience, and the results reported here should not depend in any way on the choice of ROIs (as can be seen from the near ubiquitous drop in standardized effects across all ROIs in Fig. 4).

HCP Emotion model. The full Bayesian mixed model for Y_{it}, representing the neural response within a particular ROI (separately standardized for each subject) for the ith subject toward the jth stimulus in the kth experimental condition at the tth time point, is

$$Y_{it} \sim \text{Normal}(\mu_{it}, \sigma_Y), \qquad \sigma_Y \sim \text{HalfCauchy}(\ 10),$$

where HalfCauchy was chosen as the default, weakly informative prior for the standard deviation (Gelman, 2006), and the mean is

$$\mu_{it} = \alpha_1 Y_{it-1} + \alpha_2 Y_{it-2} + \beta_0 + \sum_{k=1}^{3} (\beta_k + p_{ki}) X_{kit} + \sum_{j=1}^{23} s_j x_{ijt}$$

Where α_1 and α_2 are fixed effects accounting for autoregressive order-2 variation; β_0 and β_k are fixed effects of the intercept and condition k, respectively; p_{ki} are the random subject effects, the disturbance from β_k for subject i, condition k; s_j are the random stimulus effect for stimulus j. The autoregressive fixed effects have Cauchy priors and the remaining fixed effects have Normal priors,

$$\alpha_1, \alpha_2 \sim \text{Cauchy}(0,1), \qquad \beta_0, \beta_k \sim \text{Normal}(0, 10^6).$$

The random subject effects are Normal with the same HalfCauchy prior

$$p_{ki} \sim \text{Normal}(0, \sigma_k), \qquad \sigma_k \sim \text{HalfCauchy}(10).$$

The stimuli random effects likewise have the same type Normal/HalfCauchy priors; for shape stimuli,

$$X_{1it} = \sum_{j=1}^{3} x_{ijt}, \qquad s_1, ..., s_3 \sim \text{Normal}(0, \sigma_{\text{Shape}}), \qquad \sigma_{\text{Shape}} \sim \text{HalfCauchy}(10).$$

For anger face stimuli,

$$X_{2it} = \sum_{j=4}^{13} x_{ijt}, \qquad s_4, ..., s_{13} \sim \text{Normal}(0, \sigma_{\text{Anger}}), \qquad \sigma_{\text{Anger}} \sim \text{HalfCauchy}(10).$$

For fear face stimuli,

$$X_{3it} = \sum_{j=14}^{23} x_{ijt}, \qquad s_{14}, ..., s_{23} \sim \text{Normal}(0, \sigma_{\text{Fear}}), \qquad \sigma_{\text{Fear}} \sim \text{HalfCauchy}(10).$$

The two contrasts of interest in this model are the Face vs. Shape contrast, $(\beta_2 + \beta_3)/2 - \beta_1$, and the Anger vs. Fear contrast, $\beta_2 - \beta_3$.

HCP Working Memory model. The full Bayesian mixed model for Y_{it} -- representing the neural response within a particular ROI (separately standardized for each subject) for the ith subject toward the jth stimulus in the kth stimulus category under the lth N-back condition at the tth time point -- has a similar form as above:

$$Y_{it} \sim \text{Normal}(\mu_{it}, \sigma_Y), \qquad \sigma_Y \sim \text{HalfCauchy}(\ 10),$$

$$\mu_{it} = \alpha_1 Y_{it-1} + \alpha_2 Y_{it-2} + \beta_0 + \sum_{k=1}^{4} \beta_k X_{kit} + \sum_{l=1}^{2} (\gamma_l + p_{li}) N_{lit} + \sum_{j=1}^{96} s_j x_{ijt},$$

$$\alpha_1, \alpha_2 \sim \text{Cauchy}(0, 1), \qquad \beta_0, \beta_k \sim \text{Normal}(0, 10^6), \qquad \gamma_l \sim \text{Normal}(0, 10^6),$$

$$p_{li} \sim \text{Normal}(0, \sigma_l), \qquad \sigma_l \sim \text{HalfCauchy}(10).$$

For this model, the stimuli are as follows; for body stimuli,

$$X_{1it} = \sum_{j=1}^{24} x_{ijt}, \qquad s_1, ..., s_{24} \sim \text{Normal}(0, \sigma_{\text{Body}}), \qquad \sigma_{\text{Body}} \sim \text{HalfCauchy}(10).$$

For place stimuli,

$$X_{2it} = \sum_{j=25}^{48} x_{ijt}, \qquad s_{25}, ..., s_{48} \sim \text{Normal}(0, \sigma_{\text{Place}}), \qquad \sigma_{\text{Place}} \sim \text{HalfCauchy}(10).$$

For face stimuli,

$$X_{3it} = \sum_{j=49}^{72} x_{ijt}, \qquad s_{49}, ..., s_{72} \sim \text{Normal}(0, \sigma_{\text{Face}}), \qquad \sigma_{\text{Face}} \sim \text{HalfCauchy}(10).$$

For tool stimuli,

$$X_{4it} = \sum_{j=73}^{96} x_{ijt}, \qquad s_{73}, ..., s_{96} \sim \text{Normal}(0, \sigma_{\text{Tool}}), \qquad \sigma_{\text{Tool}} \sim \text{HalfCauchy}(10).$$

The regressors X_{1it} to X_{4it} were constructed in the usual way (by convolution with a double-gamma HRF) and then orthogonalized with respect to N_{1it} and N_{2it}, which were the regressors of interest. Each in the series of 24 trials consists of both 1- and 2-back stimuli. N_{1it} and N_{2it} represent the predicted neural responses for the 0-back and 2-back trials, respectively, of the N-back task used in the HCP Working Memory data. Note that both the participants and the stimuli were crossed with the two N-back conditions, i.e., all subjects made responses in both conditions and all stimuli received responses under both the conditions. The contrast of interest in this model is the difference in response between the 0-back and 2-back conditions, $\gamma_1 - \gamma_2$.

HCP Social Cognition model. The full Bayesian mixed model for Y_{it}, representing the neural response within a particular ROI (separately standardized for each subject) for the ith subject toward the jth stimulus in the kth experimental condition at the tth time point, has the form:

$$Y_{it} \sim \text{Normal}(\mu_{it}, \sigma_Y), \qquad \sigma_Y \sim \text{HalfCauchy}(\ 10),$$

$$\mu_{it} = \alpha_1 Y_{it-1} + \alpha_2 Y_{it-2} + \beta_0 + \sum_{k=1}^{2} (\beta_k + p_{ki}) X_{kit} + \sum_{j=1}^{10} s_j x_{ijt},$$

$$\alpha_1, \alpha_2 \sim \text{Cauchy}(0, 1), \qquad \beta_0, \beta_k \sim \text{Normal}(0, 10^6),$$

$$p_{ki} \sim \text{Normal}(0, \sigma_k), \qquad \sigma_k \sim \text{HalfCauchy}(10).$$

The stimuli are as follows; for the random stimuli,

$$X_{1it} = \sum_{j=1}^{5} x_{ijt}, \qquad s_1, ..., s_5 \sim \text{Normal}(0, \sigma_{\text{Random}}), \qquad \sigma_{\text{Random}} \sim \text{HalfCauchy}(10).$$

For the mental stimuli:

$$X_{2it} = \sum_{j=6}^{10} x_{ijt}, \qquad s_6, ..., s_{10} \sim \text{Normal}(0, \sigma_{\text{Mental}}), \qquad \sigma_{\text{Mental}} \sim \text{HalfCauchy}(10).$$

The contrast of interest in this model is the random vs. mental contrast, $\beta_1 - \beta_2$.

Chang *et al.* (2015) model. For this dataset we used a slightly simplified, approximate version of the full RSM, quite similar to what Gelman & Hill (2007) refer to as a “no-pooling model.” The basic idea is that rather than directly modeling all of the neural data for all subjects for a particular ROI at once, one first fits separate subject-level models that simply contain a separate fixed regressor for each stimulus. Since the participants and stimuli are fully crossed in this dataset, this produces $n \times m$ estimated regression coefficients, where *n* is the number of subjects and *m* is the number of stimuli. These estimated regression coefficients are then propagated upward to serve as the data in a higher-level RSM that includes crossed random participant and stimulus effects. This model is only an approximate version of the full RSM, as it ignores the varying degrees of uncertainty in the stimulus-level coefficients estimated at the first level (due in particular to factors such as differences in how often the stimuli were presented and how collinear the stimulus-level regressors were for each subject). We used this approximate method because this is the form in which the data were made available on the public NeuroVault repository (NeuroVault.org; Gorgolewski *et al*., 2015). However, it is worth noting that two practical advantages of this simplified model are that (i) it can be estimated relatively quickly and efficiently compared to the full RSM, and (ii) it can be fit using standard mixed modeling software such as R’s lme4 package or SAS PROC MIXED, since the higher-level model is just a straightforward crossed random effects model with one and only one stimulus effect associated with each data point.

For the statistical details of the first-level model, which was applied separately to each subject’s neural data, see Chang *et al.* (2015). From these first-level models one obtains $n \times m$ coefficients b_{ij}, representing the estimated regression coefficient for the neural response of the ith subject toward the jth stimulus. After standardizing the coefficients separately for each subject, the 2nd level model is then, as before, the Normal/HalfCauchy observation model

$$b_{ij} \sim \text{Normal}(\mu_{ij}, \sigma_Y), \qquad \sigma_Y \sim \text{HalfCauchy}(\ 10),$$

with mean given by

$$\mu_{ij} = \beta_0 + s_j + (\beta_1 + p_i)X_{ij},$$

where now β_0 is the mean response to all stimuli, s_j is the random stimulus effect, β_1 is the fixed effect of valence, p_i is the random subject effect of valence, and X_{ij} is a dummy variable indicating whether the jth stimulus photograph was negative or neutral in valence. The fixed variables have wide Normal priors,

$$\beta_0, \beta_1 \sim \text{Normal}(0, 10^6),$$

And the random effects have the Normal/HalfCauchy priors as before:

$$s_j \sim \text{Normal}(0, \sigma_S), \qquad \sigma_S \sim \text{HalfCauchy}(10),$$
$$p_i \sim \text{Normal}(0, \sigma_P), \qquad \sigma_P \sim \text{HalfCauchy}(10).$$

OpenfMRI Emotion Regulation model. The full Bayesian mixed model for Y_{it}, representing the neural response within a particular ROI (separately standardized for each subject) for the ith subject toward the jth stimulus in the kth experimental condition at the tth time point, takes the form of the time-series models above:

$$Y_{it} \sim \text{Normal}(\mu_{it}, \sigma_Y), \qquad \sigma_Y \sim \text{HalfCauchy}(\ 10)$$
$$\mu_{it} = \alpha_1 Y_{it-1} + \alpha_2 Y_{it-2} + \beta_0 + \sum_{k=1}^{3}(\beta_k + p_{ki})X_{kit} + \sum_{j=1}^{120} s_{kj} x_{kijt}$$
$$\alpha_1, \alpha_2 \sim \text{Cauchy}(0, 1), \qquad \beta_0, \beta_k \sim \text{Normal}(0, 10^6),$$
$$p_{ki} \sim \text{Normal}(0, \sigma_k), \qquad \sigma_k \sim \text{HalfCauchy}(10).$$

For the Negative/Suppress stimuli,

$$X_{1it} = \sum_{j=1}^{80} x_{1ijt}, \qquad s_{1,1}, \ldots, s_{1,80} \sim \text{Normal}(0, \sigma_{\text{Neg/S}}), \qquad \sigma_{\text{Neg/S}} \sim \text{HalfCauchy}(10).$$

For the Negative/Attend stimuli,

$$X_{2it} = \sum_{j=1}^{80} x_{2ijt}, \qquad s_{2,1}, \ldots, s_{2,80} \sim \text{Normal}(0, \sigma_{\text{Neg/A}}), \qquad \sigma_{\text{Neg/A}} \sim \text{HalfCauchy}(10).$$

For the Neutral/Attend stimuli,

$$X_{3it} = \sum_{j=81}^{120} x_{3ijt}, \qquad s_{3,81}, \ldots, s_{3,120} \sim \text{Normal}(0, \sigma_{\text{Neu/A}}), \qquad \sigma_{\text{Neu/A}} \sim \text{HalfCauchy}(10).$$

Note that all of the negative stimuli ($j = 1, \ldots, 80$) are observed in both the Suppress and Attend conditions, while the neutral stimuli ($j = 81, \ldots, 120$) are observed only in the Attend condition, so that there are a total of 200 stimulus effects (80+80+40) for the 120 stimuli. The two contrasts of interest in this model are the Negative vs. Neutral contrast, $(\beta_1 + \beta_2)/2 - \beta_3$, and the Suppress vs. Attend contrast, $\beta_1 - (\beta_2 + \beta_3)/2$. Although we report only the Negative vs. Neutral results in the main text for the sake of brevity, the results for the Suppress vs. Attend contrast were nearly identical in terms of the impact of the RSM on standardized effects; standardized effects in the RSM were reduced by about 14% on average compared to those from the standard model.

HCP Language model. This dataset consisted of subjects' responses toward 6 stories and 831 math problems (drawn from a much larger potential set of over 7,000 unique stimuli). These math problems were generally presented a very small number of times each--most appeared only once in the dataset, and many appeared only twice. Due to the intensive computation that would have been required to estimating models with 831 extra parameters (most of which were either not identifiable or only weakly identifiable), we decided to group all of the math stimuli with fewer than 3 responses into a single "dummy" stimulus, resulting in a new total of 272 math stimuli. The full Bayesian mixed model for Y_{it}, representing the neural response within a particular ROI (separately standardized for each subject) for the ith subject toward the jth stimulus in the kth experimental condition at the tth time point, takes the same form as the previous model:

$$Y_{it} \sim \text{Normal}(\mu_{it}, \sigma_Y), \qquad \sigma_Y \sim \text{HalfCauchy}(\ 10),$$

$$\mu_{it} = \alpha_1 Y_{it-1} + \alpha_2 Y_{it-2} + \sum_{k=1}^{2} (\beta_k + p_{ki}) X_{kit} + \sum_{j=1}^{272} s_j x_{ijt},$$

$$\alpha_1, \alpha_2 \sim \text{Cauchy}(0, 1), \qquad \beta_k \sim \text{Normal}(0, 10^6),$$

$$p_{ki} \sim \text{Normal}(0, \sigma_k), \qquad \sigma_k \sim \text{HalfCauchy}(10).$$

For the story stimuli,

$$X_{1it} = \sum_{j=1}^{6} x_{ijt}, \qquad s_1, ..., s_{10} \sim \text{Normal}(0, \sigma_{\text{Story}}), \qquad \sigma_{\text{Story}} \sim \text{HalfCauchy}(10).$$

For the math stimuli,

$$X_{2it} = \sum_{j=11}^{272} x_{ijt}, \qquad s_{11}, ..., s_{272} \sim \text{Normal}(0, \sigma_{\text{Math}}), \qquad \sigma_{\text{Math}} \sim \text{HalfCauchy}(10).$$

The contrast of interest in this model is the Story vs. Math contrast, $\beta_1 - \beta_2$.

Simulation

We simulated experiments in which n participants respond one time each to m stimuli that belong to one of two stimulus categories, and the contrast of interest is the difference in neural activation between the two categories. The simulated experiments used a block design with the stimuli presented in alternating blocks of 8, with the order of the stimulus category presentations (i.e., whether the sequence began with category A or category B) counterbalanced across participants. Each experimental run consisted of an initial stimulus presented for 1 second, then a 2-second inter-stimulus interval (ISI), then another stimulus presented for 1 second, then another 2-second ISI, and so on until all stimuli had been presented once. The stimulus regressors x_{ijt} and X_{kit} were formed in the same way as described in the previous "Statistical models" section.

The data Y_{it}, representing the neural response within a particular voxel or ROI for the ith subject toward the jth stimulus in the kth experimental condition at the tth time point, were generated according to the model

$$Y_{it} \sim \text{Normal}(\mu_{it}, \sigma_Y), \qquad \sigma_Y = 1,$$

where the mean is given by

$$\mu_{it} = \alpha_1 Y_{it-1} + \alpha_2 Y_{it-2} + \sum_{k=1}^{2} (\beta_k + p_{ki}) X_{kit} + \sum_{j=1}^{m} s_j x_{ijt}.$$

The autocorrelation parameters were set equal to

$$\alpha_1 = .45, \qquad \alpha_2 = .15,$$

and the fixed means for the two stimulus categories were set equal to

$$\beta_1 = 1, \qquad \beta_2 = 2.$$

The random participant effects p_{ki} and the random stimulus effects $s)j$ were distributed as

$$p_{ki} \sim \text{Normal}(0, 1), \qquad s_j \sim \text{Normal}(0, \sigma_{\text{Stim}}).$$

In our simulations we varied the number of participants $n \in \{16, 32, 64\}$; the number of stimuli $m \in \{16, 32, 64\}$; and the standard deviation of the random stimulus effects $\sigma_{\text{Stim}} \in \{0, 1, 2\}$. We ran 500 iterations of the simulation within each of these parameter combinations. In each iteration of the simulation we fit 4 statistical models to the simulated dataset, which we describe next.

SPM model. The first model that we fit to each simulated dataset is perhaps the most widely used statistical model in neuroimaging, serving as the default model in the SPM software package. In practice it is implemented in a two-stage procedure. In the first stage, one fits separate regression models to the data from each participant, where the model for the ith participant is

$$Y_{it} = \alpha_{1i} Y_{it-1} + \alpha_{2i} Y_{it-2} + \sum_{k=1}^{2} \beta_{ki} X_{kit} + e_{it}, \qquad e \sim \text{Normal}(0, \sigma_i).$$

In the second stage, one computes the difference $\hat{\beta}_{1i} - \hat{\beta}_{2i}$ for each participant and then performs a one-sample t-test on these differences. This model can be seen as an approximation to a mixed-effects model with random participant effects -- what we refer to below as the No Stimulus Model (NSM) and in the main text as the standard model -- valid to the extent that the intrasubject variances are all equal. We include the results of this model in our simulations simply to show that it yields practically equivalent results to the NSM model that we focus on in the main text, despite their slight differences in assumptions. In the main text we focus on comparing the Random Stimulus Model (RSM) to the NSM, rather than the SPM model, because it is a cleaner comparison, since the RSM and NSM are identical except for their inclusion of random stimulus effects.

Fixed stimulus model (FSM). The second model that we fit is similar in structure to the RSM, except the stimuli are treated as fixed effects rather than random effects. In other words, a separate fixed regression coefficient is estimated for each individual stimulus regressor. This model has been described by (Mumford, Turner, Ashby, & Poldrack, 2012), who referred to it as the "Least Squares -- All" model. Specifically, the FSM that we estimated in each iteration is

$$Y_{it} \sim \text{Normal}(\mu_{it}, \sigma_Y), \qquad \sigma_Y \sim \text{HalfCauchy}(\ 10),$$

$$\mu_{it} = \alpha_1 Y_{it-1} + \alpha_2 Y_{it-2} + \sum_{k=1}^{2} p_{ki} X_{kit} + \sum_{j=1}^{m} \beta_j x_{ijt},$$

$$\alpha_1, \alpha_2 \sim \text{Cauchy}(0, 1), \qquad \beta_j \sim \text{Normal}(0, 10^6),$$

$$p_{ki} \sim \text{Normal}(0, \sigma_k), \qquad \sigma_k \sim \text{HalfCauchy}(10).$$

Note that the random stimulus effects s_j are replaced with fixed effects β_j, which also requires that the category fixed effects β_1 and β_2 are removed from the model, since they would be perfectly collinear with the fixed stimulus effects. The contrast between the two stimulus categories is computed as the mean of the β_j in stimulus category B minus the mean of the β_j in stimulus category A.

No stimulus model (NSM). This is the model that we referred to in the main text as the "standard model." Unlike the FSM and RSM, the NSM does not include any stimulus effects at all, hence the name we use here. Specifically, the NSM that we estimated in each iteration is:

$$Y_{it} \sim \text{Normal}(\mu_{it}, \sigma_Y), \qquad \sigma_Y \sim \text{HalfCauchy}(\ 10),$$
$$\mu_{it} = \alpha_1 Y_{it-1} + \alpha_2 Y_{it-2} + \sum_{k=1}^{2} (\beta_k + p_{ki}) X_{kit},$$
$$\alpha_1, \alpha_2 \sim \text{Cauchy}(0, 1), \qquad \beta_k \sim \text{Normal}(0, 10^6),$$
$$p_{ki} \sim \text{Normal}(0, \sigma_k), \qquad \sigma_k \sim \text{HalfCauchy}(10).$$

The contrast between the stimulus categories was computed as $\beta_2 - \beta_1$.

Random stimulus model (RSM). The RSM that we estimated in each iteration is:

$$Y_{it} \sim \text{Normal}(\mu_{it}, \sigma_Y), \qquad \sigma_Y \sim \text{HalfCauchy}(\ 10),$$
$$\mu_{it} = \alpha_1 Y_{it-1} + \alpha_2 Y_{it-2} + \sum_{k=1}^{2} (\beta_k + p_{ki}) X_{kit} + \sum_{j=1}^{m} s_j x_{ijt},$$
$$\alpha_1, \alpha_2 \sim \text{Cauchy}(0, 1), \qquad \beta_k \sim \text{Normal}(0, 10^6),$$
$$p_{ki} \sim \text{Normal}(0, \sigma_k), \qquad \sigma_k \sim \text{HalfCauchy}(10).$$

For stimulus category A,

$$s_1, ..., s_{m/2} \sim \text{Normal}(0, \sigma_A), \qquad \sigma_A \sim \text{HalfCauchy}(10).$$

For stimulus category B,

$$s_{m/2+1}, ..., s_m \sim \text{Normal}(0, \sigma_B), \qquad \sigma_B \sim \text{HalfCauchy}(10).$$

The contrast between the stimulus categories was computed as $\beta_2 - \beta_1$.

False positive rates under the SPM model

We first present the results for the false positive rates (i.e., Type 1 error rates) of the SPM model in a separate simulation identical to the main simulation, but with $\beta_1 = \beta_2 = 1$, so that the null hypothesis of no difference in the fixed category means is true. These error rates are based on running 500 iterations for each parameter combination, and they are computed for several levels of α, the decision threshold. Test levels of α= 0.05, 0.01, 0.005 and 0.001 are considered, which have nominal Monte Carlo 95% confidence intervals of ± 0.019, ± 0.009, ± 0.006, and ± 0.003, respectively.

			**alpha = .05**	**alpha = .01**	**alpha = .005**	**alpha = .001**

sigma_stim	n	m				
**0**	**16**	**16**	0.068	0.014	0.004	0.002
		**32**	0.054	0.014	0.002	0.002
		**64**	0.044	0.006	0.006	0.000
	**32**	**16**	0.060	0.012	0.010	0.000
		**32**	0.060	0.006	0.004	0.000
		**64**	0.062	0.012	0.008	0.002
	**64**	**16**	0.052	0.020	0.010	0.004
		**32**	0.056	0.014	0.010	0.000
		**64**	0.044	0.008	0.002	0.000
**1**	**16**	**16**	0.178	0.058	0.036	0.008
		**32**	0.128	0.056	0.036	0.010
		**64**	0.118	0.038	0.028	0.010
	**32**	**16**	0.262	0.158	0.124	0.062
		**32**	0.224	0.102	0.080	0.036
		**64**	0.130	0.042	0.024	0.008
	**64**	**16**	0.442	0.312	0.260	0.182
		**32**	0.342	0.196	0.142	0.072
		**64**	0.248	0.130	0.102	0.050
**2**	**16**	**16**	0.344	0.190	0.148	0.082
		**32**	0.274	0.150	0.106	0.046
		**64**	0.208	0.104	0.080	0.030
	**32**	**16**	0.478	0.316	0.270	0.200
		**32**	0.438	0.322	0.262	0.170
		**64**	0.290	0.186	0.148	0.080

	**64**	**16**	0.642	0.548	0.514	0.410
		**32**	0.568	0.442	0.400	0.310
		**64**	0.452	0.298	0.254	0.184

Inflation of standardized effects

The plots below show the simulated standardized effects for the contrast between the two stimulus categories for all four models in each parameter combination. The points in each panel give the mean standardized effects across 500 simulation runs, and the error bars span plus-or-minus one standard deviation of the standardized effects across the 500 simulation runs. It is worth noting that the parameter estimates we obtained for all three of the Bayesian models described above were not discernibly affected by the particular choice of priors. While we initially experimented with more informative priors (e.g., modeling stimulus or subject variances as $\sim \mathrm{HalfCauchy}(1)$) , the insensitivity of the posterior estimates to the choice of prior ultimately led us to opt for the relatively weak, or uninformative, priors detailed above. The interpretation of these simulation results is given in the Online Methods section in the main text. The full simulation results for all parameters of all models under all parameter combinations can be found at http://github.com/PsychoinformaticsLab/nipymc (Yarkoni and Westfall, 2016).

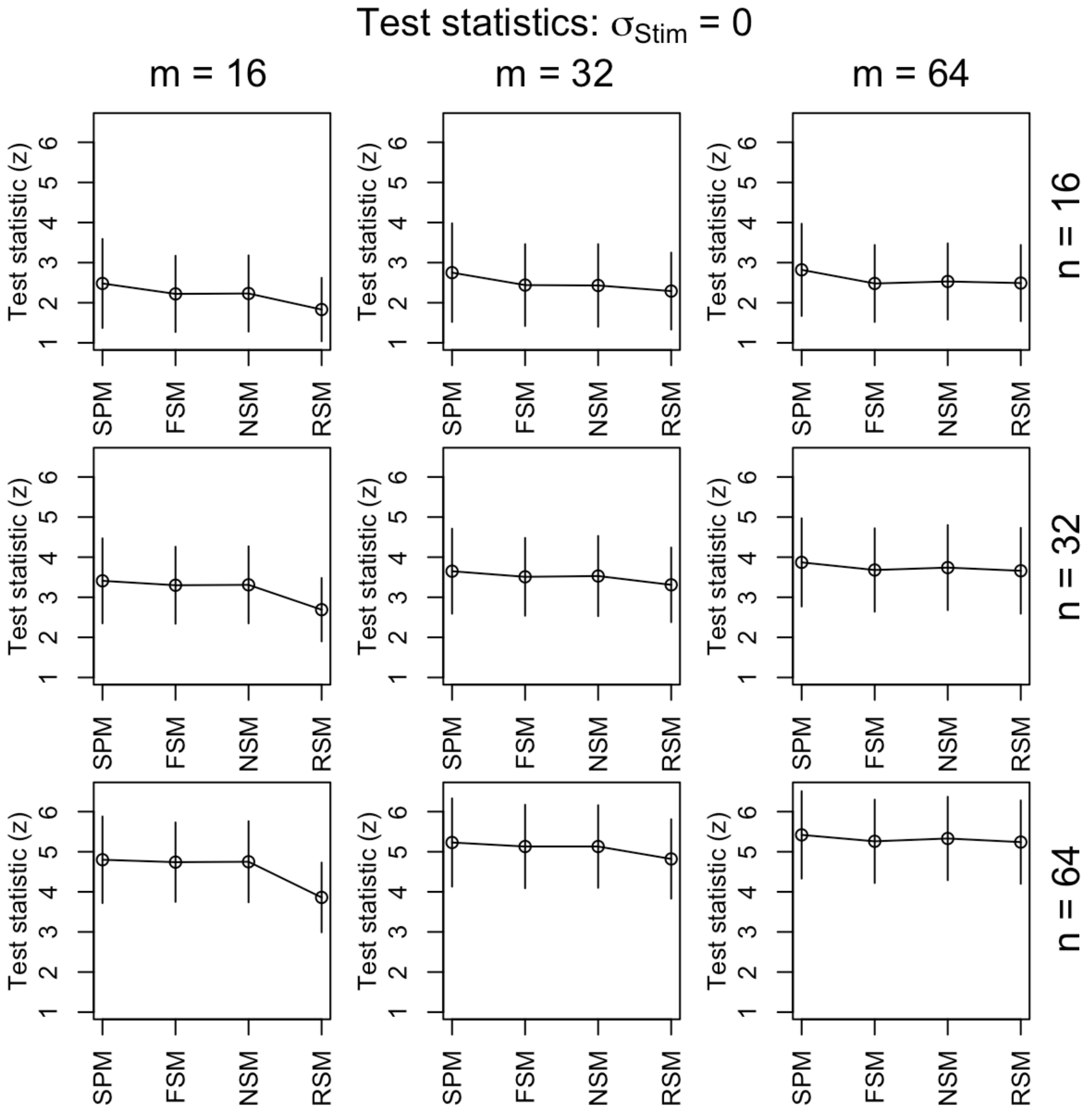

Test statistics: σ_{Stim} = 0
m = 16
m = 32
m = 64
n = 16
n = 32
n = 64
Test statistic (z)
SPM
FSM
NSM
RSM

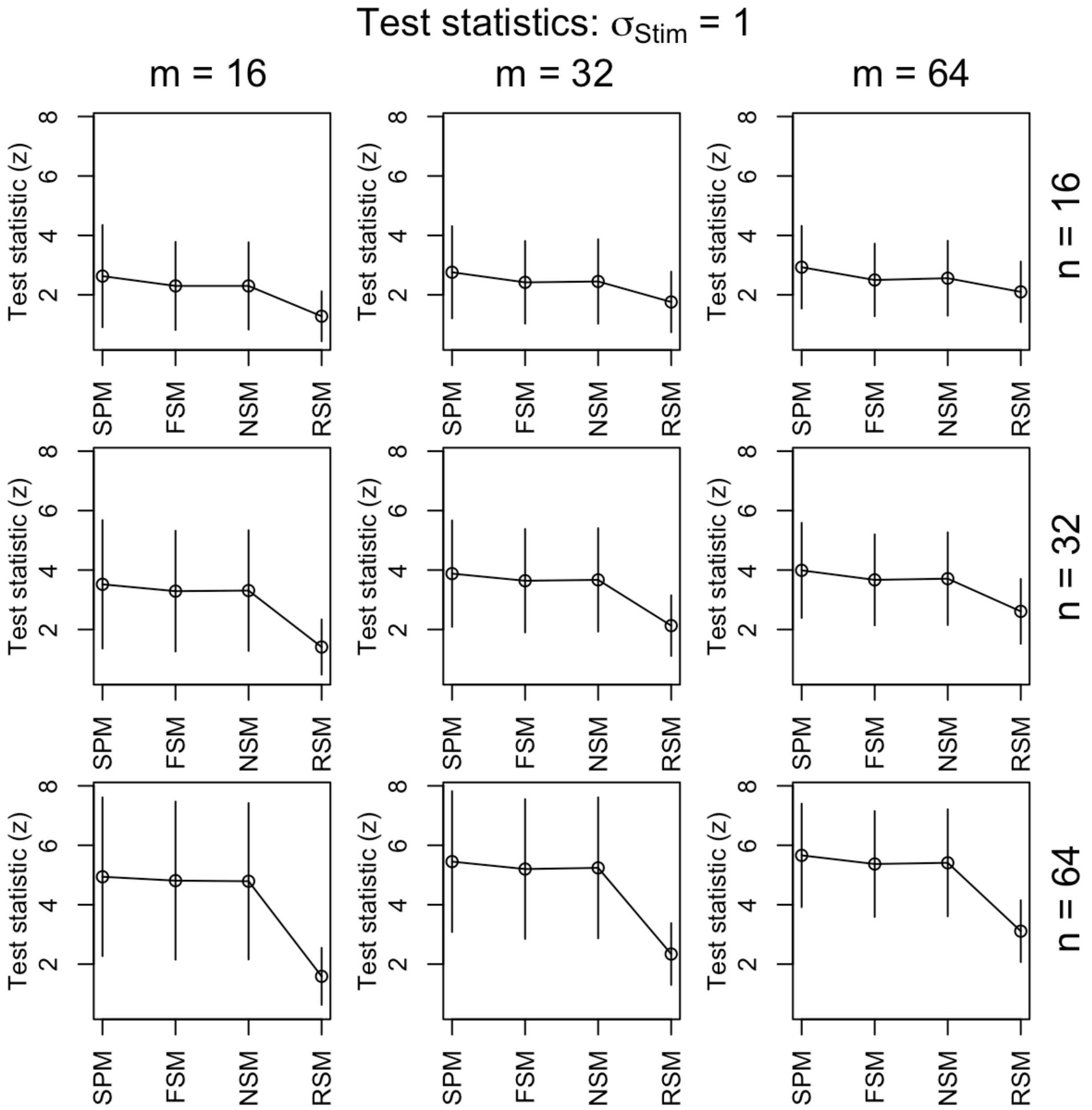
Test statistics: σ_{Stim} = 1
m = 16
m = 32
m = 64
n = 16
n = 32
n = 64
Test statistic (z)
SPM
FSM
NSM
RSM
2
4
6
8

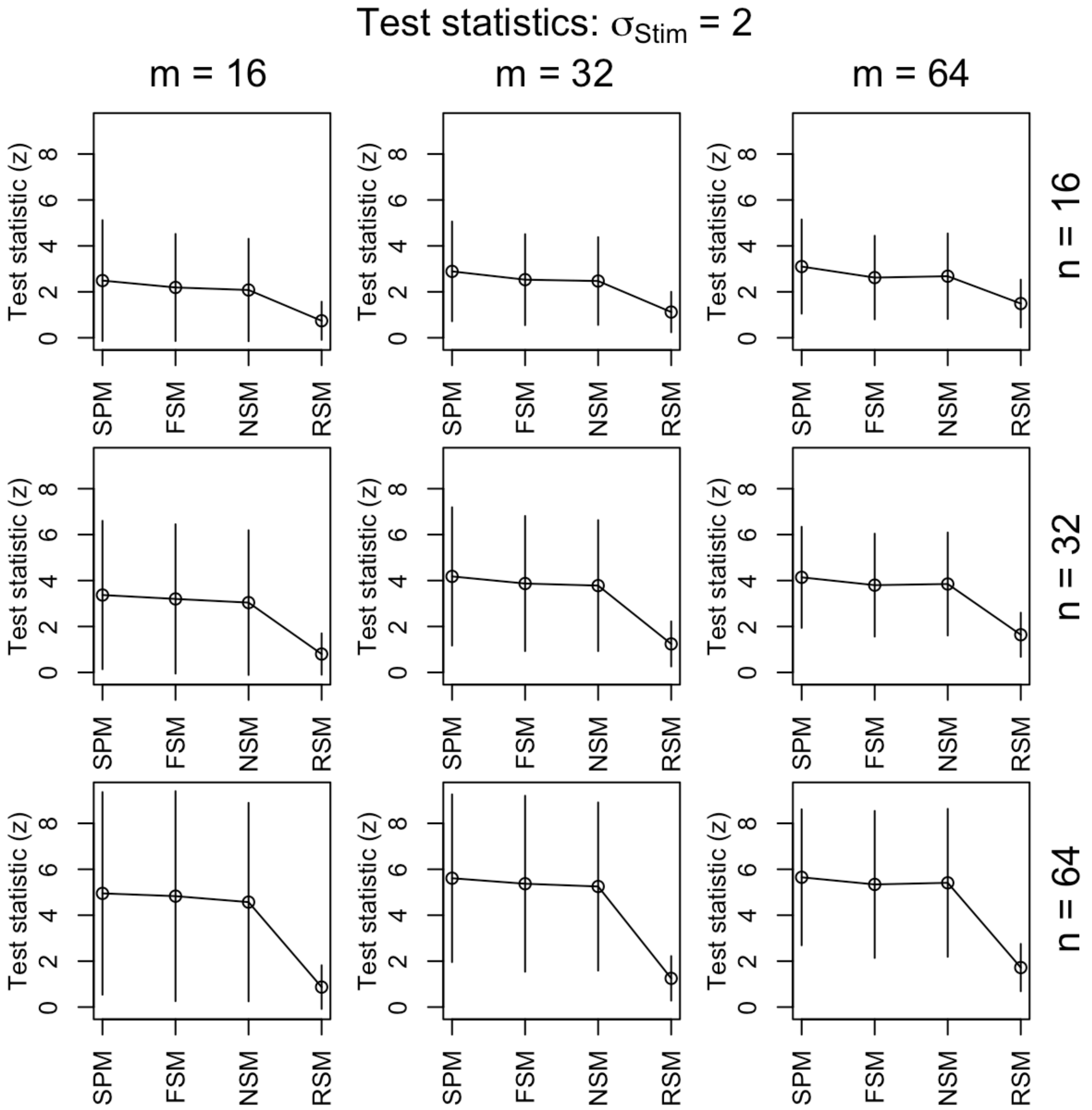

Test statistics: σ_{Stim} = 2
m = 16
m = 32
m = 64
n = 16
n = 32
n = 64
Test statistic (z)
0
2
4
6
8
SPM
FSM
NSM
RSM

Additional references

Gelman, A. (2006). Prior distributions for variance parameters in hierarchical models (Comment on Article by Browne and Draper). *Bayesian Analysis*, *1*(3), 515–534.

Mumford, J. A., Turner, B. O., Ashby, F. G., & Poldrack, R. A. (2012). Deconvolving BOLD activation in event-related designs for multivoxel pattern classification analyses. *NeuroImage*, *59*(3), 2636–2643.

Yarkoni, T and Westfall J (2016). PsychoinformaticsLab/nipymc: v0.0.1-alpha [Dataset]. *Zenodo*. DOI, 10.5281/zenodo.168087.
