# Supplementary material for: Fixing the stimulus-as-fixed-effect fallacy in task fMRI
Source: Wellcome Open Res. 2017 Mar 17;1:23. Originally published 2016 Dec 9. [Version 2] doi: 10.12688/wellcomeopenres.10298.2 (PMC5428747; doi:10.12688/wellcomeopenres.10298.2)
Supplement: Peer review history

# Peer review: Referee response for version 2

**DOI:** 10.21956/wellcomeopenres.11977.r21482

**Published:** 2017-05-10

**Authors:** Michael B. Miller, Benjamin O. Turner

**Affiliations:** 1 Department of Psychological & Brain Sciences, University of California Santa Barbara, Santa Barbara, CA, USA

In the present article, Westfall and colleagues point out a major oversight in nearly all existing GLM fMRI analyses that attempt to make a generalization about stimuli —namely the failure to treat stimuli as random effects. Far from being a technical shortcoming with little practical consequence, the authors demonstrate that the failure to account for stimulus variance can markedly (and spuriously) inflate inferential statistics, with the worst inflation occurring with large numbers of participants and small numbers of unique stimuli. The authors present an analytic solution to this problem, as well as recommending a number of design precepts that can ameliorate the problem even while still using the standard fixed-effect analysis approach. This is an important issue, which is particularly timely given growing concerns in the field about reproducibility, and the case the authors make is compelling. However, the manuscript left us with a number of outstanding questions.

 We think it would be helpful to the readers to point out that most fMRI studies are not looking to make generalizations about classes of stimuli. For example, a memory researcher will typically use a fixed set of stimuli (e.g., faces or words) for study. But the stimuli are typically counter-balanced or randomly assigned across experimental conditions (e.g., studied items or non-studied items) and across subjects, in which case, modeling random effects for the stimuli is not necessary. It’s only in limited cases in which generalizations about stimuli are being claimed (e.g., greater activity for nouns compared to verbs) that it becomes necessary. The authors do mention in the introduction that a “review of 100 random task-based fMRI article extracted from the Neurosynth database . . . used multiple stimuli in a context where generalizations over stimuli was clearly indicated.” [the authors indicated that details about this procedure could be found in the supplementary materials, but we could not find them] However, this strikes us as not representative of published fMRI studies. In any case, we think the authors should make it clear that the concerns they raise only apply to those studies that make generalizations about classes of stimuli.

 The biggest issue that was not addressed in the current manuscript is how the stimulus-as-fixed-effect issue impacts analytic approaches other than the standard mass univariate GLM. (Indeed, the authors occasionally seem to ignore the existence of other approaches, as when they claim there is a “universal failure of fMRI studies” to acknowledge stimulus variability.) For example, it would seem that many applications of representational similarity analysis (RSA; Kriegeskorte, Mur, & Bandettini, 2008 ^1^) are not affected by this issue. Relatedly, it is not uncommon for researchers to include various parametric regressors that may vary across stimuli within the same class; does such an approach do anything to address the stimulus-as-fixed-effect issue, and conversely, is the authors’ proposed RSM approach compatible with the use of parametric regressors?

 Similarly, a recent report by Abdulrahman and Henson ^2^ addressed the question of variability, not just at the stimulus level, but at the trial level. Indeed, although that paper was primarily concerned with multivariate approaches, they somewhat surprisingly found that trial-level methods (namely, the “least-squares separate” and “least-squares all” methods of Mumford, Turner, Ashby, & Poldrack, 2012 ^3^) could sometimes outperform traditional approaches even in the context of the GLM. Westfall and colleagues should explain whether (and if so, how) the stimulus-as-fixed-effect issue impacts alternative analysis approaches like RSA and the use of parametric regressors, and whether they would expect trial-level methods to play any role in ameliorating (or exacerbating) the issue.

 There were also some details which were glossed over or not reported, which would be very useful to readers. Some of these might require additional work, but others should be readily available from what the authors have done already. For example, the discussion of the simulation results was very cursory. Likewise, although the authors allude to the computational demands of their RSM approach, it would be useful to quantify exactly what these demands are, for example by providing an estimate of how long some fixed number of analyses, involving some number of stimuli or number of TRs, takes on a particular system with particular specs. Among information that might require additional analyses but would be useful to researchers, the most pressing need relates to defining what “completely unique stimulus set” means—that is, is there some parametric relationship between the degree to which each participant’s stimulus set is unique and how suboptimal the standard fixed-effect approach is? E.g., in a study of 30 participants, is it unique enough if each participant sees a random subset of 99% of the stimuli in some larger corpus? 75%? 50%?

 The last concern has to do with being careful in defining the scope of the proposed RSM approach. While the entire paper reflects the high degree of importance the authors place on drawing valid inferences, we nonetheless felt that the section “Stimulus-level parameter estimates as a tool for explanation” should emphasize the need for caution to an even greater degree than it already does. This is a particularly exciting possible application of the approach, but it is also one that seems particularly prone to abuse, especially by researchers who are looking for an upside to counteract the “downside” of smaller effect sizes that this approach will bring. The authors are obviously statistically sophisticated enough that they leave implicit the dangers associated with this sort of exploratory approach; they should be explicit.

We have read this submission. We believe that we have an appropriate level of expertise to confirm that it is of an acceptable scientific standard, however we have significant reservations, as outlined above.

## References

[1] KriegeskorteNMurMBandettiniP: Representational similarity analysis - connecting the branches of systems neuroscience. *Front Syst Neurosci*.2008;2: 10.3389/neuro.06.004.2008 4 10.3389/neuro.06.004.2008 19104670PMC2605405

[2] AbdulrahmanHHensonRN: Effect of trial-to-trial variability on optimal event-related fMRI design: Implications for Beta-series correlation and multi-voxel pattern analysis. *Neuroimage*.2016;125: 10.1016/j.neuroimage.2015.11.009 756-66 10.1016/j.neuroimage.2015.11.009 26549299PMC4692520

[3] MumfordJATurnerBOAshbyFGPoldrackRA: Deconvolving BOLD activation in event-related designs for multivoxel pattern classification analyses. *Neuroimage*.2012;59(3) : 10.1016/j.neuroimage.2011.08.076 2636-43 10.1016/j.neuroimage.2011.08.076 21924359PMC3251697

# Peer review: Referee response for version 2

**DOI:** 10.21956/wellcomeopenres.11977.r21481

**Published:** 2017-04-19

**Authors:** Robert Leech, Romy Lorenz

**Affiliations:** 1 Division of Brain Sciences, Computational, Cognitive and Clinical Neuroimaging Laboratory (C3NL), Imperial College London, London, UK

This is a very well-written and easy to follow paper, convincingly demonstrating the fallacy of not modelling stimulus variability. The paper conveys an important message, which has been completely overlooked in the neuroimaging field to date and is likely to become influential over time, especially as practical guidelines are provided and the code has been made publicly available.

 We have a number of queries and suggestions detailed below:

**Main points:**

**Introduction**

 There appear to be two related issues in the paper: one is not modelling structured noise in the data, inflating standardised effects; this is the case when comparing the standard model which doesn’t model stimulus variability to the RSM and other models. This may be wrong even in the situation where the researcher was agnostic as to whether the results apply in general to categories of stimuli or just the stimuli tested. The second issue is fixed versus random effect modelling of the stimuli which is presented as the main focus of the paper. If this is the case, it might be worth making the distinction clearer.

 It would be beneficial to introduce to the reader that you will focus on a particular example in your result sections, e.g., amygdala response to emotional faces; it was slightly surprising to us when we approached the Results section.

**Methods**

 In the simulations, is stimulus-to-stimulus variability kept constant across subjects? The ground truth of this isn’t known, but is this plausible? What is the effect of relaxing this, is it similar to increasing the number of stimuli tested? How does it interact with the number of participants and inflation of standardised effects? It would be helpful to justify this and consider its implications in the discussion.

 P.2 | Section fMRI dataset: Please list the tasks and specific contrasts you chose from the HCP dataset to be included in your analyses as it seems that you focused on a sub-selection (Figure 4 and Figure 6). While all information are mentioned in the text, it might be clearer to create a table summarizing sample size and number of stimuli for each dataset (not just HCP) or include this information in the two figures, n=Y, and m=X.

 P. 5| Section Simulations: You mention that you fitted four different models, which are also explained in detail in the Supplementary File you provide. However, it would be helpful to state in the main text that the standard model, the fixed stimulus model, and the random stimulus model are implemented as Bayesian models to facilitate comparison (as differences in results can be solely linked to the inclusion of no, fixed, or random stimulus effects in the model), while the SPM model is used to compare results against the main software in the field (but here the implementations differs).

**Results**

 Some explicit details about the computational demand for model fitting would be interesting to provide.

**Discussion**

 The empirical studies used for comparison had sample sizes N=100, N=72, and N=11. The study with the small sample size (N=11) however included many different stimuli types (M=60). In this respect, no study was included that had a sample size commonly found in the field (N=20-30) while also having a relatively small number of stimuli as also common in the field as outlined in your Introduction. This kind of study though would have been the best when making inferences about a “large fraction of the results reported in the fMRI literature”. Considering that the problem of reduction in standardized effects is the highest for studies with large sample sizes, the conclusion that a “large fraction of the results reported in the fMRI literature are likely to be severely inflated” (p.11) may be too strong given the empirical studies employed for this study.

 You may want to discuss that when the data contained zero stimulus variability, the RSM model yielded attenuated effects compared to the standard model (by 18%) for small stimulus sample size. While this may not be of practical relevance (as zero stimulus variability is highly unlikely), it would be important to reflect on this from a statistical point of view.

 Are there other similar effects that might be affecting the FMRI literature? For example, what about stimulus presentation order? Both cognitive and neural processes likely have a large systematic temporal effect. Despite having large influence on the BOLD signal, this is normally not modelled, and so could also affect standardised effect sizes and interpretation. It may not be comparable, but it might be worth speculating on other potential problems, which could have similar effect, such as temporal order, in the discussion.

 Currently, you take a statistician’s point of view as the main focus of the paper. However, one potential implication of the work that could be discussed is that stimuli selection based on classic psychology theory may be not completely accurate. For example, while it could be that psychology informed stimuli selection would group stimulus A and stimulus B in the same category, they may not be represented in the brain that way; the  neural system may be  more sensitive to stimuli perturbations than could be assessed using 'traditional' psychological tools (derived from questionnaire or behavioural data). Equally, different representations of stimuli in the brain could result in similar behavioural responses. This points towards the need for a neurally-informed cognitive taxonomy rather than relying purely on psychological definitions, that were developed largely blind to the underlying functional organisation of the brain.**

 Minor points/typos

- P.2 : When referring to the literature review, please also point to the Method section besides the Supplementary File 1 as it may not be clear to the reader that methodological details about the simulations are provided in the method section of the main text and not in Supplementary File 1.
- P.4 | Section Simulations: You are referring to the Appendix I where it should state Supplementary File 1 instead.
- P.5 | Section Literature review: Please refer that main results were presented in Introduction and more details are provided in Supplementary File 1. Otherwise, readers may miss results of this analysis in the Result section of the main paper.
- P. 5 | Section Simulations: Please refer to Supplementary File 1 as overview of results is provided there.
- P.2-5: Please consider re-ordering the subsections in the Method section. From a reader’s point of view, we would suggest the following order: Statistical Modeling, Simulations, fMRI datasets, Literature Review
- P.9:Missing closing bracket and full stop at end of first paragraph, i.e., (cf. Figure 2).

We have read this submission. We believe that we have an appropriate level of expertise to confirm that it is of an acceptable scientific standard, however we have significant reservations, as outlined above.

# Peer review: Referee response for version 2

**DOI:** 10.21956/wellcomeopenres.11977.r21045

**Published:** 2017-03-28

**Authors:** Sophie Donnet

**Affiliations:** 1 University of Paris-Saclay, Paris, France

The authors mainly answered to my comments.

I have read this submission. I believe that I have an appropriate level of expertise to confirm that it is of an acceptable scientific standard.

# Peer review: Referee response for version 1

**DOI:** 10.21956/wellcomeopenres.11091.r19186

**Published:** 2017-01-20

**Authors:** Sophie Donnet

**Affiliations:** 1 University of Paris-Saclay, Paris, France

In this paper, the authors claim that the conclusions of several studies would be modified if a statistical models taking into account the variability of the presented stimuli had been considered. 

 Although the problem is interesting, I am not completely convinced by the conclusions and the statistical tools used to assess the results. 

 First of all, the authors argue that, due to the complexity of the new model, the authors can not use the standard numerical tools to perform the statistical inference (R or SAS) and so will prefer a Bayesian inference, making this choice quite opportunist. However, besides the fact that they use a Bayesian inference (including prior distribution), they base their conclusions on frequentist arguments (comparing test statistics). To my point of view, this is quite confusing. If a Bayesian framework is considered, then the hypothesis testings should be perform using Bayes Factor or any other tools taking into account the prior distribution.

 Moreover, when proposing a new model, the first thing to do is to  compare the old model and the new model for any given dataset (using statistical tools such as hypothesis testing, in a frequentist or Bayesian framework). I could not see such a test in the paper. The mixed effects model involves much more parameters for the same amount of observations. Before deriving conclusions on the new model, it should be interesting to put in competition the fixed effects model and the mixed effects models, to be sure that the use of the more complex model is supported by the data.

 Besides, in a Bayesian context, the greater posterior variance observed on the regression parameters was completely expected (Figure 2) (more parameters for the same quantity of information leads to more incertitude a posteriori). However, without objective criteria (such as Bayes factor or hypothesis testing), I am not able to decide whether the difference between fear faces and anger faces is significant or not. (caption for Figure 2). It is not clearly stated in the paper how the authors were able to do so (even though I could find clues in the simulation section?).  

 I am aware of the fact that Bayes factor are quantities difficult to estimate and that frequentist hypothesis testing in mixed effects models can be a tough issue. However, if the authors want to prove that the conclusions of statistical testing are modified when using mixed effects models, then they should perform the adequate and rigorous statistical testings.

I have read this submission. I believe that I have an appropriate level of expertise to state that I do not consider it to be of an acceptable scientific standard, for reasons outlined above.

# Response

**Published:** 2017-03-06

**Authors:** Thomas Nichols

**Affiliations:** University of Warwick, UK

We’d like to thank Dr. Donnet for taking the time to consider our paper carefully. For ease of reading we've pasted in her comments as boldface, bulleted text.

- **In this paper, the authors claim that the conclusions of several studies would be modified if a statistical models taking into account the variability of the presented stimuli had been considered. **
- **Although the problem is interesting, I am not completely convinced by the conclusions and the statistical tools used to assess the results. **
- **First of all, the authors argue that, due to the complexity of the new model, the authors can not use the standard numerical tools to perform the statistical inference (R or SAS) and so will prefer a Bayesian inference, making this choice quite opportunist.**

We regret that the choice to use a Bayesian model created confusion.  We noted in the Methods section that the fMRI random stimulus model “cannot be fit using standard mixed modeling statistical packages, such as lme4 in R or SAS PROC MIXED, because these packages assume that each row of the dataset is associated with one and only one level of each random factor, i.e., a single participant and a single stimulus.” Hence, we were forced to develop a custom model, and a Bayesian one developed with PyMC3 was the most convenient approach.

 We note that most of the datasets we analyzed could in principle also have been fitted using a modified (and approximate) version of the standard “summary statistics” model; however, this would have required extensive additional analysis, as we would have needed to fit a separate first-level model for each individual subject (adding individual regressors for each individual stimulus and temporarily omitting existing condition regressors, which in the classical formulation are non-identifiable in the presence of the stimulus effects); and then fit a mixed model at the second level that models the individual stimulus effects as random effects. The former steps would have been extremely time-consuming relative to our strategy of fitting a single large model, and the latter step (i.e., modeling stimulus as a random effect) is currently not supported in any existing fMRI package, so we would have had to develop a custom model anyway. Moreover, as we noted in the supplement, “This [summary statistics] model is only an approximate version of the full RSM, as it ignores the varying degrees of uncertainty in the stimulus-level coefficients estimated at the first level (due in particular to factors such as differences in how often the stimuli were presented and how collinear the stimulus-level regressors were for each subject)”.

 We also note that we did in fact use the summary statistics approximation to obtain estimates for the Chang et al. (2015) dataset (see supplement for details). This was not by choice, and we would have preferred to fit the full RSM; however, as we explain in the supplement, this dataset was available in a form amenable only to the summary statistics approach (i.e., because pre-computed estimates for each individual trial were provided).

- **However, besides the fact that they use a Bayesian inference (including prior distribution), they base their conclusions on frequentist arguments (comparing test statistics). To my point of view, this is quite confusing.**

We do not believe that our conclusions are based on frequentist arguments. Notably, we have not computed any p-values nor refer to any z-scores.  In the section “Does the amygdala preferentially respond to emotional faces?” we write: “we define a test statistic z = µ/σ, where µ and σ are the mean and standard deviation, respectively, of the posterior samples for the associated parameter estimate”. The use of “test statistic” was a bit careless, as we are not “testing hypotheses”; we simply needed a convenient summary of the posterior.  We admit that this choice of terminology could be confusing, so in our revision we have renamed this statistic the posterior standardized effect.

 Another important point is that our argument is fundamentally qualitative and not quantitative; we focus on how uncertainty surrounding the estimate of interest (denominator of our standardized effect) increases dramatically in most cases. We present this increase in terms of standardized effect because that’s what most people are familiar with, but nothing would change if we instead talked about the relative increases in the variances of the estimates.

- **If a Bayesian framework is considered, then the hypothesis testings should be perform using Bayes Factor or any other tools taking into account the prior distribution.**

Bayes Factors in this setting are not trivial, and any rate would not serve the broad audience, who would like to know:  How severe is the random stimulus effect problem?  Again, we could have measured this directly in terms of variance inflation, but since users are most familiar with signal-to-noise ratio, we felt that this was the best metric.

- **Moreover, when proposing a new model, the first thing to do is to  compare the old model and the new model for any given dataset (using statistical tools such as hypothesis testing, in a frequentist or Bayesian framework). I could not see such a test in the paper. The mixed effects model involves much more parameters for the same amount of observations. Before deriving conclusions on the new model, it should be interesting to put in competition the fixed effects model and the mixed effects models, to be sure that the use of the more complex model is supported by the data.**

The reviewer is correct that no formal model assessment was used. This was intentional, as (crucially) we are not testing a hypothesis about the relative fit of different models.  Rather, we’re pointing out a fundamental mismatch between the conclusions researchers typically draw (which almost invariably involve assuming that it is safe to generalize conclusions over a population of stimuli) and the inferences that are actually licensed by the statistical model (which, in the absence of a variance component reflecting the random sampling of levels from some factor, are only valid for the specific levels used in the experiment). Our position is that it is the design of the experiment and the goals of the analyst--not the observed data--that determine which random effects should be included in the model. This position is consistent with the arguments of Barr, Levy, Scheepers, and Tily (2013).

 It may be useful to draw an analogy to a random subject effect in a multi-subject model.  No reasonable investigator would test for the significance (or BF-based evidence) for a subject random effect; while such tests may come up negative sometimes, we know our subjects are a random sample from a larger population, a population to which we wish to generalise.  Likewise, it is now generally accepted that meta-analyses should treat study as a random effect irrespective of formal tests of heterogeneity (Borenstein, et al., 2010).

 Hence our paper is not focused on the question “Is there statistical evidence for random stimulus effects?” but rather the assertion “Some stimuli are random, and such stimuli should be so modeled.”  This is a widely noted point in many other disciplines, as we have referenced in the introduction; our contribution is to raise awareness of this issue in neuroimaging, and provide easy-to-interpret measures of the impact of neglecting random stimulus effects.

- **Besides, in a Bayesian context, the greater posterior variance observed on the regression parameters was completely expected (Figure 2) (more parameters for the same quantity of information leads to more incertitude a posteriori).**

Our reanalysis of the Emotion Regulation dataset and our simulation results both speak to the contrary. The Emotion regulation dataset featured a relatively large number of stimuli with small estimated stimulus variance; consequently the standardized effects (what we called the test statistics in the manuscript) from the RSM and standard models are essentially identical, despite the fact that the RSM technically contains many more parameters. Consistent with this general finding, in the Method subsection describing our simulation results we wrote: “When the true data generating process contained zero stimulus variability, the standard model and RSM yielded very similar [standardized effects] for the stimulus category difference, for all participant and stimulus sample sizes. The exception was that when the stimulus sample size was small (m = 16), the [standardized effects] from the RSM were slightly attenuated (by about 18%) compared to the standard model [standardized effects]. However, this attenuation disappeared with increasing stimulus sample size, so that at m = 32 and m = 64, the [standardized effects] from the two models were essentially identical, as should be the case given the lack of true stimulus variability.” Note that as the stimulus sample size increases, the number of parameters in the RSM technically increases as well, but this actually causes the posterior for the category difference to become more narrow, not more wide. We believe that these analyses sufficiently highlight the conditions under which the RSM does and does not give overly pessimistic posterior estimates of the category difference.

- **However, without objective criteria (such as Bayes factor or hypothesis testing), I am not able to decide whether the difference between fear faces and anger faces is significant or not. (caption for Figure 2). It is not clearly stated in the paper how the authors were able to do so (even though I could find clues in the simulation section?).  **

We were careful not to claim that the difference was or was not statistically significant, but rather, that there appears to be at most a small effect. In Bayesian estimation terms, this amounts to saying that the posterior distributions for the effects of fearful and angry faces mostly overlap. We have updated the text to clarify these points (see updated section beginning with “Depending on one’s statistical approach and assumptions…”).

- **I am aware of the fact that Bayes factor are quantities difficult to estimate and that frequentist hypothesis testing in mixed effects models can be a tough issue. However, if the authors want to prove that the conclusions of statistical testing are modified when using mixed effects models, then they should perform the adequate and rigorous statistical testings.**

As expanded on above, we do not think model comparison is necessary or helpful in this context. Our argument is not predicated on the idea that a model with random stimulus effects fits the data better (although we strongly suspect that it often will), but rather on how the experimental design and the desired inferences demand such a model.

 REFERENCES

 Barr, D. J., Levy, R., Scheepers, C., & Tily, H. J. (2013). Random effects structure for confirmatory hypothesis testing: Keep it maximal. Journal of Memory and Language, 68(3). https://doi.org/10.1016/j.jml.2012.11.001

 Borenstein, M., Hedges, L. V., Higgins, J. P. T., & Rothstein, H. R. (2010). A basic introduction to fixed-effect and random-effects models for meta-analysis. Research Synthesis Methods, 1(2), 97–111. http://doi.org/10.1002/jrsm.12
